# Synthesis of a Dual-Color Fluorescent Dendrimer for Diagnosis of Cancer Metastasis in Lymph Nodes

**DOI:** 10.3390/polym14204314

**Published:** 2022-10-14

**Authors:** Chie Kojima, Kento Nagai

**Affiliations:** Department of Applied Chemistry, Graduate School of Engineering, Osaka Prefecture University, 1-1, Gakuen-cho, Naka-ku, Sakai 599-8531, Osaka, Japan

**Keywords:** dendrimer, dual color, fluorescence imaging, lymph node, tumor cells

## Abstract

Detection of cancer metastasis spread in lymph nodes is important in cancer diagnosis. In this study, a fluorescence imaging probe was designed for the detection of both lymph node and tumor cells using always-ON and activatable fluorescence probes with different colors. Rhodamine B (Rho), a matrix metalloproteinase−2 (MMP−2)-responsive green fluorescence probe, and a tumor-homing peptide were conjugated to a carboxy-terminal dendrimer that readily accumulates in lymph nodes. The activatable green fluorescence signal increased in the presence of MMP−2, which is secreted by tumor cells. Both the always-ON Rho signal and the activatable green fluorescence signal were observed from tumor cells, but only the weak always-ON Rho signal was from immune cells. Thus, this type of dendrimer may be useful for non-invasive imaging to diagnose cancer metastasis in lymph nodes.

## 1. Introduction

Bioimaging is a valuable non-invasive tool that can be used in the diagnosis of diseases such as cancer. Many bioimaging techniques have been developed, including magnetic resonance imaging, nuclear medicine imaging, and fluorescence imaging [1,2,3]. Fluorescence imaging is a widely used technique that is versatile in allowing observation at different magnifications to image small objects such as cells to the entire bodies of small animals. In fluorescence imaging, both intensity and wavelength are detected. As multi-color fluorescence imaging yields a multitude of information, it has been used for purposes such as the mapping of lymph nodes [1,2,3,4]. Fluorescence probes can be categorized as always-ON and activatable ones. As the signal-to-noise ratio is quite important in imaging, always-ON probes with targeting ability are useful for achieving detection with high selectivity. In contrast, activatable fluorescence probes, where a signal is activated in response to a specific stimulus, are useful for achieving detection with high sensitivity [5,6]. Activatable fluorescence probes have been studied for use in the detection of cancer. For example, a matrix metalloprotease−2 (MMP−2) substrate peptide combined with a quencher and a fluorescent agent at both ends has been synthesized [7]. This probe does not exhibit fluorescence emission in the absence of MMP−2 via a fluorescence resonance energy transfer (FRET) process that takes place from the fluorophore to the quencher. However, the possibility of FRET occurring can be eliminated via peptide cleavage in the presence of MMP−2, resulting in fluorescence emission. As MMP−2 is involved in the invasion, metastasis, and angiogenesis of cancer, it is a cancer marker [8]. The MMP−2-responsive fluorescence probe can thus be used to detect cancer cells [7].

As metastasis is the main reason for a reduced survival rate from cancer, the diagnosis of this process is thus extremely important. Cancer cells often metastasize from a primary tumor to the lymph nodes around tumor tissues. The sentinel lymph node (SLN) is the first lymph node that cancer cells have metastasized from the primary tumor reach. As the presence of tumor cells in the SLN is currently examined via biopsy during surgical treatment [9,10], it is thus desirable to develop non-invasive approaches that can be used before surgeries. Besides, the preservation of lymph nodes is essential, as lymph node-resident immune cells play important roles in cancer immunotherapy [11,12]. Although various imaging probes have been studied for use in SLN imaging and mapping [4,11,12,13,14,15,16,17], there are only a few reports on systems that allow the visualization of tumor cells in lymph nodes [18,19,20].

Dendrimers are highly branched synthetic polymers that have a controllable structure. Dendrimers can be loaded with various types of bioactive molecules, such as drugs, imaging probes, and ligands, via either or both their conjugation to the terminal functional groups and encapsulation in an internal cavity of dendrimers. Thus, dendrimers are po-tent multifunctional nanocarriers for use in nanomedicine [1,21,22,23,24]. Previously, we developed dendrimers for delivery to lymph nodes and elucidated that the terminal structure of the dendrimer largely affects the association with immune cells in the lymph nodes. Anionic carboxy-terminal polyamidoamine (PAMAM) dendrimers of larger than generation 4 (G4) have been shown to accumulate in lymph nodes after intradermal injection without being recognized by immune cells, including macrophages [25,26,27]. We previously designed a carboxy-terminal dendrimer conjugated to an MMP−2-responsive fluorescent component and a tumor-homing peptide as an imaging probe to visualize lymph node-resident tumor cells [28]. The roles of the carboxy-terminal dendrimer, the MMP−2-responsive green fluorescence probe, and the tumor-homing peptide are that they can be delivered into a lymph node, detect cancer cells, and associate with cancer cells, respectively. This imaging probe can thus be used to visualize lymph nodes with metastatic tumor cells, but not those with non-metastatic tumor cells. In this study, the carboxy-terminal dendrimer with the MMP−2-responsive green fluorescence probe and tumor-homing peptide was modified with an always-ON red fluorescent probe to produce a dual-color fluorescent dendrimer. As lymph nodes and metastatic tumor cells can be visualized from the always-ON red fluorescence and activatable green fluorescence, respectively, this dendrimer is thus useful for distinguishing between lymph nodes that have/do not have metastatic tumor cells at different colors using fluorescence imaging. Herein, the design and synthesis of the dendrimer were described, and the MMP−2 responsiveness of the dendrimeric imaging probe was shown. In addition, fluorescence imaging of metastatic tumor cells and immune cells was performed using the dendrimeric imaging agent.

## 2. Materials and Methods

### 2.1. Synthesis

The carboxy-terminal dendrimer bearing Rho (always-ON probe), *p*MR–pep–FITC (activatable fluorescence probe), and tLyp-1 (tumor-homing peptide) were synthesized, as follows. First, an amino-terminal PAMAM dendrimer (G4) with an ethylenediamine (EDA) core was purchased from Sigma–Aldrich Co. LLC. (St. Louis, MO, USA). To this, four equivalents of rhodamine B isothiocyanate (Rho ITC) (Sigma–Aldrich, 17 mg, 32 μmol) were reacted with the PAMAM dendrimer (113 mg, 8 μmol) in dimethyl sulfoxide (DMSO) at room temperature for 8 h. After its subsequent dialysis, the dendrimer was obtained in 62% yield after freeze-drying.

The subsequent reactions were carried out, as described in our previous work [25,26,27,28], in which the Rho-conjugated dendrimer was reacted with excess succinic anhydride in an aqueous alkaline solution to obtain the carboxy-terminal Rho-conjugated dendrimer [25,28]. *p*MR–pep–FITC and tLyp-1 were used, which were synthesized in our previous report [28]. *p*MR–pep–FITC was conjugated to the Rho-conjugated carboxyl-terminal dendrimer via an EDA linker [27,28], and tLyP-1 was conjugated to the carboxyl-terminal dendrimer bearing Rho and *p*MR–pep–FITC via a 2-maleimidoethylamine linker [28].

### 2.2. Characterization

The dendrimer was dissolved in deuterated water containing deuterated sodium hydroxide for recording proton nuclear magnetic resonance (^1^H NMR) spectroscopy measurements using a 400 MHz JNM-LA 400 JNM-ECX spectrometer (JEOL Co., Ltd., Tokyo, Japan), as described in our previous work [25,26,27,28].

The dendrimer was dissolved in DMSO, and the UV-vis spectra (450–650 nm) of the dendrimer solution was measured using an ultraviolet-visible (UV-vis) spectrophotometer (V-630, JASCO, Tokyo, Japan). The peptide bound number was estimated from the standard curves of Rho and pMR–pep–FITC, as described in our previous work [28].

The fluorescamine assay was performed, as described in our previous work [28]. Briefly, 2 mg/mL of fluorescamine solution (200 μL, acetone) was added to 0.4 mg/mL of dendrimer solution (0.5 M borate buffer (pH 8.5)), whereafter the fluorescence intensity of the solution was measured at 479 nm after excitation at 390 nm using an FP-6200 spectrofluorometer (JASCO). The fluorescence intensity of the dendrimer before the addition of fluorescamine was subtracted from that after the addition, and then the amount of tLyp-1 was estimated from its standard curve.

### 2.3. MMP−2 Assay

The MMP−2 assay was performed, as previously described in the literature [28]. Briefly, 1 μM of a solution of the synthesized dendrimer was digested with 0.5 μg/mL of MMP−2 (catalytic domain, human, recombinant, Enzo Life Sciences, Inc., Farmingdale, NY, USA) in a buffer containing 50 mM of Tris, 10 mM of CaCl_2_, and 150 mM of NaCl (pH 7.4) after the pre-incubation of MMP−2 at 37 °C for 15 min. Emission spectra in the range of 490–600 nm (ex 488 nm) were recorded over 24 h using an FP-6200 spectrofluorometer.

### 2.4. In Vitro Fluorescence Imaging

In vitro fluorescence imaging was performed, as previously described in the literature [28]. HT-1080 and RAW264 cells were obtained from the Japanese Collection of Research Bioresources Cell Bank (Osaka, Japan) and RIKEN Cell Bank (Ibaraki, Japan), respectively, which were maintained in Eagle’s minimal essential medium with non-essential amino acids containing 10% fetal bovine serum. Each cell line (6 × 10^3^ cells) was seeded on a 96-well plate and cultured at 37 °C for 24 h. Then, the synthesized dendrimer was added (3 μM for each dye). After incubation for 4 h, the cells were washed with phosphate-buffered saline (PBS) and then observed using an inverted fluorescence microscope (ECLIPSE Ti-U, Nikon Corp., Tokyo, Japan) equipped with a super high-pressure mercury lamp and a microscopy camera (WRAYCAM-SR300, WRAYMER Inc., Osaka, Japan).

## 3. Results and Discussion

### 3.1. Design and Synthesis of the Dendrimeric Dual-Color Fluorescent Probe

**Figure 1:**
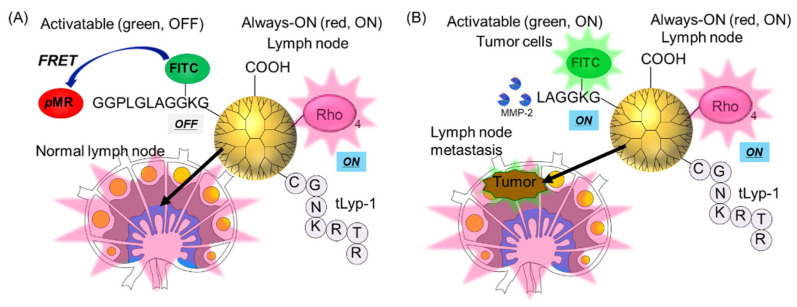
Design of a dual-color fluorescent carboxyl-terminal dendrimer featuring a tumor-homing peptide (tLyp-1), rhodamine B (Rho) as an always-ON probe, and a matrix metalloprotease−2 (MMP−2) substrate peptide combined with a quencher (*p*MR) and a fluorophore (FITC) at both ends as an activatable fluorescence probe. Using the probe, single- and dual-color fluorescence signals can be observed from normal lymph node (**A**) and metastatic tumor cells-containing lymph node (**B**), respectively.

The dendrimeric dual-color fluorescence probe was designed using a carboxyl-terminal PAMAM dendrimer, Rho as an always-ON probe, an MMP−2-responsive green fluorescence probe, and tLyp-1 as a tumor-homing peptide, as shown in Figure 1. The carboxyl-terminal dendrimer accumulates in lymph nodes without being recognized by various immune cells [25,26,27], making it a possible nanoplatform to deliver the imaging probe into lymph nodes. As tLyP-1 (CGNKRTR) is known to be internalized in cancer cells via neuropilin-1 and neuropilin-2 [29,30], the addition of tLyP-1 to the dendrimer induces cellular uptake in tumor cells. The MMP−2-responsive green fluorescence probe comprises an MMP−2 substrate peptide (GGPLGLAGGKG) with *p*-methyl red (*p*MR; absorption: 380–550 nm) at its N-terminus as a quencher and fluorescein isothiocyanate (FITC; excitation at 495 nm, emission at 520 nm) at the side chain of lysine as a fluorophore [28]. This acts as an activatable fluorescence probe that is useful for detecting tumor cells. Thus, the carboxyl-terminal dendrimer conjugated tLyp-1 and MMP−2-responsive green fluorescence probe can be used to detect tumor cells in lymph nodes [28]. Besides, the always-ON probe, Rho, was added to the dendrimer to allow the visualization of the location of the dendrimer even under conditions where green fluorescence emission is not exhibited in the absence of tumor cells.

**Figure 2:**
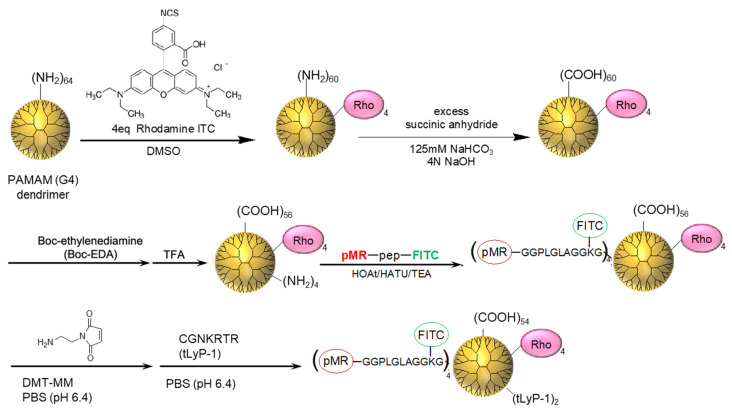
Synthesis of the dual-color fluorescent carboxyl-terminal dendrimer featuring a tumor-homing peptide. TFA, trifluoroacetic acid; HOAt, 1-hydroxy-7-azabenzotriazole; HATU, 1-[bis(dimethylamino)methylene]-1H-1,2,3-triazolo[4,5-b]pyridinium 3-oxide hexafluorophosphate; TEA, trimethylamine; DMT-MM, 4-(4,6-dimethoxy-1,3,5-triazin-2-yl)-4-methylmorpholinium chloride.

Figure 2 shows the synthetic procedure of the dual-color dendrimeric imaging probe. First, a commercially available amino-terminal PAMAM dendrimer was reacted with Rho ITC. The bound number of Rho dye was estimated to be approximately four from the UV-vis spectrum of the dendrimer solution using the standard curve for Rho (Figure S1). Then, all the residual amino groups at the dendrimer termini were converted into carboxyl groups by reacting them with succinic anhydride, the success of which was confirmed by ^1^H NMR spectroscopy (Figure S2A) [25,28]. Next, *tert*-butoxycarbonyl (Boc)-protected EDA (Boc–EDA) was reacted with the carboxyl-terminal dendrimer. The bound number of Boc–EDA was then estimated as being approximately four from the ^1^H NMR spectrum (Figure S2B) [27,28]. After the deprotection of the Boc groups at the dendrimer termini, the carboxyl-terminal group of the MMP−2-responsive fluorescence probe (*p*MR–pep–FITC) was conjugated [28]. The removal of the Boc groups was confirmed by ^1^H NMR spectroscopy (Figure S2C), and the bound number of *p*MR–pep–FITC was estimated by UV-vis spectroscopy (Figure S3). The standard curve of *p*MR–pep–FITC was recorded at 469 nm, the wavelength of the maximum absorbance, via UV-vis spectroscopy [28]. As the Rho-conjugated dendrimer showed a slight absorption at 469 nm before the conjugation of *p*MR–pep–FITC, the bound number of *p*MR–pep–FITC was calculated by subtracting the absorbance of the Rho-conjugated dendrimer without *p*MR–pep–FITC from that of the dendrimer with *p*MR–pep–FITC. Approximately four *p*MR–pep–FITC were bound to the dendrimer. Finally, the tumor-homing peptide, tLyP-1, was conjugated to the dual-color fluorescent dendrimer via a 2-maleimidoethylamine linker [28]. The bound number of tLyP-1 to the dendrimer was estimated to be approximately two by the fluorescamine assay, which is a quantitative method used to assess amino groups [28]. Consequently, a carboxy-terminal dendrimer bearing Rho (always-ON probe), *p*MR–pep–FITC (activatable probe), and tLyp-1 (tumor-homing peptide) was obtained.

### 3.2. MMP−2-Responsive Property of the Dendrimer

**Figure 3:**
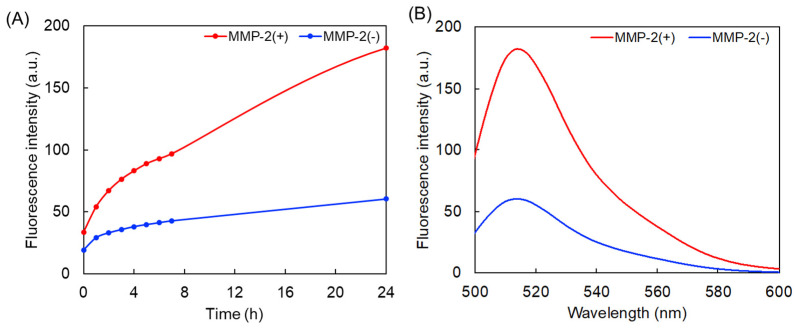
MMP−2-responsiveness of the dendrimer. (**A**) Time-dependent fluorescence intensity of the dendrimer (1 μM) at 514 nm in the absence and presence of MMP−2. (**B**) Fluorescence spectra after 24 h in the absence and presence of MMP−2.

The MMP−2-responsive property of the synthesized dendrimer was examined by measuring the green fluorescence intensity of the dendrimer in the absence and presence of MMP−2. Figure 3 shows the gradual increase in the intensity of the fluorescence signal of the dendrimer for 24 h in the presence of MMP−2. This result suggests that the dendrimer exhibits fluorescence when the MMP−2 substrate peptide is cleaved by MMP−2, upon which the expected FRET process does not occur. Our previous work showed similar results and indicated that a control peptide probe with a different sequence did not show any changes in fluorescence intensity after treatment with MMP−2 [28]. Thus, it was confirmed that the MMP−2-responsive green fluorescence probe was not suppressed by the always-ON Rho dye of the dendrimer.

### 3.3. In Vitro Fluorescence Imaging

**Figure 4:**
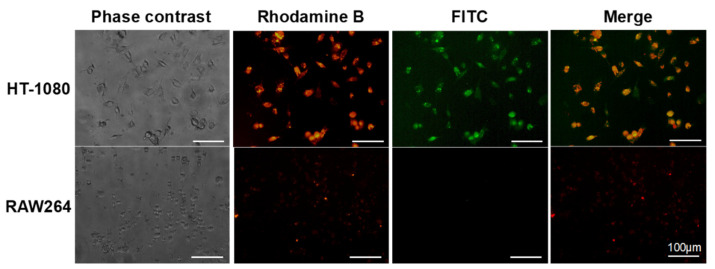
Phase contrast and fluorescence microscopic images of HT-1080 and RAW264 cells treated with the synthesized dendrimer.

In vitro fluorescence imaging of tumor and immune cells was performed using the synthesized dendrimer. HT-1080 (human fibrosarcoma cells) and RAW264 (mouse macrophage-like cells) cell lines were used as model cells. MMP−2 and neuropilin-1, a target of tLyp-1, are highly expressed in HT-1080 cells, but not in RAW264 cells [31,32,33,34]. The green fluorescence signal of the MMP−2-responsive activatable fluorescence probe was observed from the HT-1080 cells, but not from the RAW264 cells, whereas the red fluorescence signal of the always-ON probe was observed from both cell lines (Figure 4). This result indicates that dual- and single-color fluorescence signals are observed from tumor and immune cells, respectively. A lower Rho fluorescence intensity was observed from the RAW264 cells than from the HT-1080 cells, because the dendrimer did not associate well with the RAW264 cells in which the expression level of neuropilin-1 is low [34]. However, it is expected that the always-ON fluorescence signal of the dendrimer is observable in tissue in which the dendrimer is accumulated, even when it is not internalized into cells. As the carboxy-terminal dendrimer can be delivered to lymph nodes [25,26,27], the dual-color dendrimeric fluorescent probe can be used to diagnose cancer metastasis in lymph nodes.

## 4. Conclusions

A novel imaging agent comprising an MMP−2-responsive green fluorescence probe and always-ON Rho-conjugated carboxy-terminal dendrimer with a tumor-homing peptide was designed and synthesized for the diagnosis of cancer metastasis in lymph nodes. The green fluorescence intensity of the activatable fluorescence component increased in the presence of MMP−2. MMP−2-secreted metastatic tumor cells treated with the prepared dendrimeric probe exhibited dual-color fluorescence signals from its activatable fluorescence and always-ON components. However, immune cells exhibited a single-color fluorescence signal from its always-ON component, although the fluorescence signal intensity was low. These results suggest that lymph nodes with and without metastatic tumor cells can be distinguished from their different fluorescence signals. Our previous work showed that the carboxy-terminal dendrimer is accumulated in lymph nodes after intradermal injection without being recognized by immune cells [25,26,27]. Therefore, the dual-color fluorescent dendrimer has the potential to be used in the diagnosis of cancer metastasis in lymph nodes. Since dyes that exhibit green fluorescence are not useful for in vivo applications due to their inability to penetrate body tissues, near-infrared dyes and the quenchers that exhibit deeper light penetration into tissues need to be developed for in vivo imaging [35]. The improvement of the dendrimer in this work is still ongoing.

## Data Availability

The data that support the findings of this study are available from the corresponding author, upon reasonable request.

## Supplementary Materials

The following supporting information can be downloaded at: https://www.mdpi.com/article/10.3390/polym14204314/s1, Figure S1: (A) UV-vis spectrum of the Rho-conjugated dendrimer (0.05 mg/mL), and (B) standard curve of Rho. Figure S2: ^1^H NMR spectra of the Rho-conjugated carboxyl-terminal dendrimer (A) before and (B) after its conjugation with Boc–EDA. (C) ^1^H NMR spectrum of the dendrimer after Boc removal. Figure S3: UV-vis spectra of the Rho-conjugated carboxyl-terminal dendrimer before and after its conjugation with *p*MR–pep–FITC.
