# Supplementary material for: Synthesis of a Dual-Color Fluorescent Dendrimer for Diagnosis of Cancer Metastasis in Lymph Nodes
Source: Polymers (Basel). 2022 Oct 14;14(20):4314. doi: 10.3390/polym14204314 (PMC9607438; doi:10.3390/polym14204314)
Supplement: Supplementary file 1 [file polymers-14-04314-s001.zip › polymers-1923507-supplementary.pdf]

Supporting information

Synthesis of a Dual-color Fluorescent Dendrimer for Diagnosis of Cancer Metastasis in Lymph Nodes

Chie Kojima*, and Kento Nagai

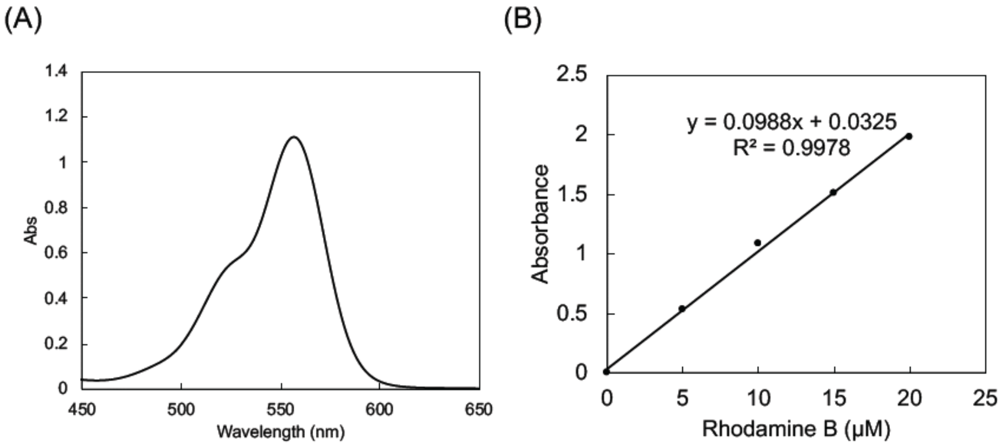

Figure S1: (A) UV–vis spectrum of the Rho-conjugated dendrimer (0.05 mg/mL). (B) Standard curve of Rho.

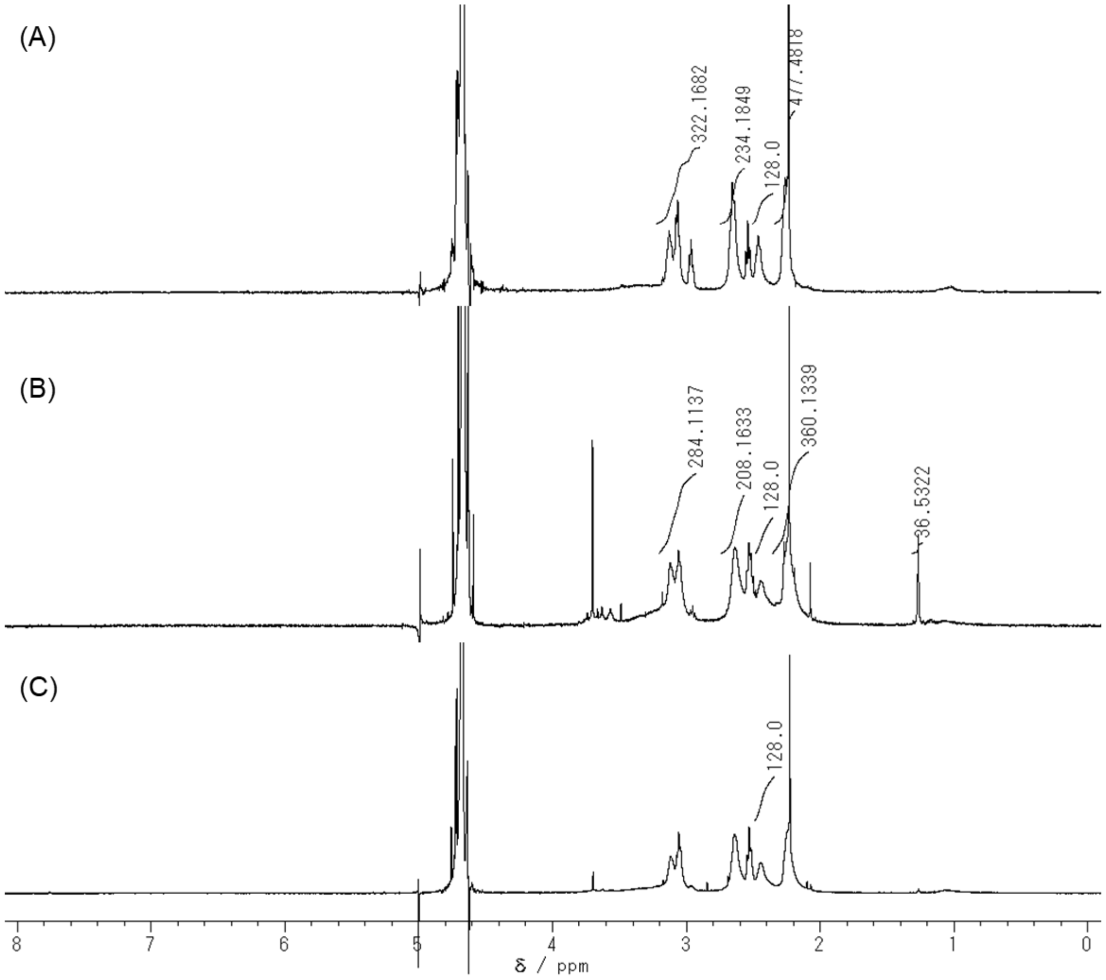

Figure S2: ^{1}H NMR spectra of the Rho-conjugated carboxyl-terminal dendrimer (A) before and (B) after its conjugation with Boc–EDA. (C) ^{1}H NMR spectrum of the dendrimer after Boc removal.

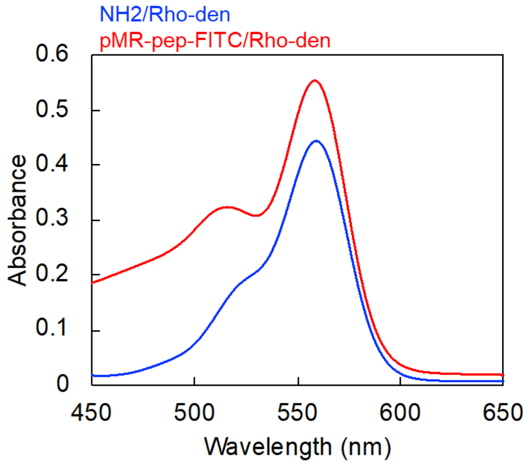

Figure S3: UV–vis spectra of the Rho-conjugated carboxyl-terminal dendrimer before and after its conjugation with *p*MR–pep–FITC.
